# Physicochemical and Sensory Properties and Antioxidant Activity of Xylitol Candies Containing Yuja (*Citrus junos*) Peels or Pulp

**DOI:** 10.3390/foods13152396

**Published:** 2024-07-29

**Authors:** Ju-Hye Im, Mi-Kyung Lee, Hae-In Lee

**Affiliations:** Food and Nutrition Department, Sunchon National University, Suncheon-si 57922, Republic of Korea; wngpp117@gmail.com (J.-H.I.); leemk@scnu.ac.kr (M.-K.L.)

**Keywords:** yuja, *Citrus junos*, xylitol candy, taste properties, antioxidant

## Abstract

Xylitol candies offer numerous health benefits such as preventing cavities and obesity. However, a preference for them tends to be low due to their distinctive flavor. In this study, we developed xylitol candies containing mature yuja peel (MYP-C), immature yuja peel (IYP-C), and yuja pulp (YP-C). To determine the optimal yuja added to xylitol candy, we compared and analyzed its physicochemical properties, sensory characteristics, and antioxidant activities. IYP-C and MYP-C significantly increased the naringin and hesperidin contents compared to the control and the YP-C. In particular, the IYP-C exhibited the highest content of flavonoids and polyphenols, which contributed to enhancing antioxidant activity such as ferric reducing antioxidant power (FRAP), 1,1 diphenyl-2-picrylhydrazyl (DPPH), and 2,2′-azino-di-2 ethyl-benzothiazoline sulfonate (ABTS+) radical scavenging activities. The IYP-C had the highest crude ash content. The L*, a*, and b* values of MYP-C and IYP-C showed dark red and yellow colors compared to the CON and YP-C groups. The sensory analysis conducted using electronic tongue equipment revealed that IYP-C exhibited high levels of umami, sweetness, and bitterness, while YP-C showed the highest intensity of sourness. In conclusion, these results suggest that IYP-C rather than MYP-C and YP-C provide xylitol candy with good qualities in terms of antioxidant activities and physicochemical characteristics.

## 1. Introduction

Sugar is one of the representative sweeteners in food that can encourage appetite, relieve exhaustion, and provide a sweet taste [1]. However, dietary sugar in excess raises the risk of various diseases such as tooth decay, obesity, and diabetes, so it should be consumed in moderation [2]. The World Health Organization (WHO) suggests that sugar intake be limited to less than 10% of gross energy to decrease the risk of unwholesome weight gain and less than 5% of gross energy to reduce dental caries development. Children and adolescents consume a lot of sugar because they prefer candy, sugar-sweetened beverages, and sugary snacks [3]. Korean children’s sugar intake is 69.6 g, which exceeds the WHO recommended intake of 57.5 g, and their sugar consumption is increasing every year due to their high demand for sweetness [4]. Excessive sugar intake in children can lead to decreased attention and concentration levels, childhood obesity, nutritional imbalance, dental caries, and childhood diabetes, so greater control is needed [4]. With increased consumer attention to reducing sugar intake, food products made with alternative sweeteners rather than sugar have become more popular [5]. Among the alternative sweeteners, xylitol has attracted the attention of consumers since it has sweetening similar to sucrose but with lower calories of 2.4 kcal/g (compared to the 4 kcal/g of typical sugars) [6]. Xylitol positively affects dental health by reducing plaque formation and preventing tooth decay, and it is effective in preventing osteoporosis by increasing calcium absorption [7,8]. Additionally, xylitol is absorbed and metabolized slowly, making it better for weight and blood sugar control than regular sugars [9]. Accordingly, xylitol has been released in various products such as candy and gum, but it has a menthol scent that some children do not like. Thus, products with various scents are being developed, such as xylitol chewing gum containing blackberry and xylitol fig yogurt [10,11].

Yuja (*Citrus junos*) is a citrus fruit mainly cultivated in Korea, Japan, etc. [12]. Citrus fruits such as oranges and grapefruits are mainly consumed for their pulp, but yuja is eaten with both the pulp and the peel, providing the advantage of easily ingesting biologically active components contained in the peel [13]. Some studies have reported that the peels of yuja fruit contain higher amounts of nutritionally valuable and biologically active components compared to the pulp [14]. Yuja peel is well known to have plenty of functional flavonoids, such as naringin and hesperidin, which have shown effects on various medicinal functions such as effects against diabetes and obesity [15,16,17]. Yuja has a strong sour taste, so it is not used as a fresh fruit but is mainly consumed in processed forms such as jam, juice, and tea, although the processing methods are not very diverse [18]. Thus, to prevent overproduction and improve the quality of yuja, immature yuja is thinned and discarded annually. However, some researchers have reported that immature yuja contains more naringin, hesperidin, dietary fiber, and pectin compared to mature yuja [19]. Therefore, to increase the usability of yuja, this study aims to develop xylitol candy containing yuja peel or pulp. Furthermore, I would like to evaluate the antioxidant, physicochemical, and sensory characteristics of a new candy product that incorporates immature yuja peel, compared to candies that use mature yuja peel and pulp.

## 2. Materials and Methods

### 2.1. Preparation of Candy

**Figure 1:**
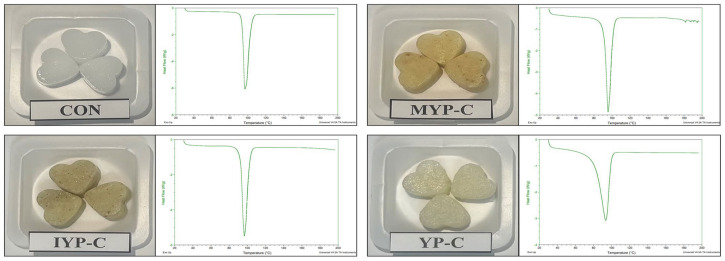
Image and DSC thermogram of xylitol candies with added yuja (*n* = 3). CON: control xylitol candy group, MYP-C: added mature yuja peel xylitol candy group, IYP-C: added immature yuja peel xylitol candy group, YP-C: added yuja pulp xylitol candy group.

The yuja used for the experiment were harvested in Goheung (Korea) in October and November 2021 and separated into pulp and peel after being washed twice with water. The yuja peel was air dried at 37 °C for 8 h, then pulverized. The yuja pulp was utilized by removing the seeds. Xylitol candies were developed under four groups: without yuja (CON), with mature yuja peel (MYP-C), with immature yuja peel (IYP-C), and with yuja pulp (YP-C), respectively (Figure 1). The 200 g of xylitol powder was placed in a container and heated at 12 °C for 20 min until the powder melted, after which the first stirring was performed. Then, heating was stopped, and the syrup was allowed to stand at room temperature for 5 min to allow the foam to settle. Once it is still, for the MYP-C sample add 20 g mature yuja peel, for the IYP-C sample add 20 g of immature yuja peel, and for the YP-C sample add 20 g yuja pulp and mix well. Then, with the help of a spoon, carefully pour the hot solution into a tray. The candies were cooled and solidified at 4 °C until completely set, individually wrapped in plastic bags, and stored in a sealed container at room temperature.

### 2.2. Analysis of Proximate Composition

The proximate composition, such as crude protein, crude lipid, ash content, moisture, and carbohydrate, of yuja candies was analyzed pursuant to Association of Official Analytical Chemists (AOAC) methods. The moisture content of the candies was determined by weight loss after 12 h of drying at 105 °C. The lipid content was determined by the Soxhlet method with a solvent extraction system (Soxtec Avanti SE-416, Gerhardt, Königswinter, Germany). The protein content was determined by the Kjeldahl method with the automatic nitrogen analyzer (VAPODEST 50S, Gerhardt, Germany). The ash content was determined according to the AOAC. The carbohydrate content was calculated by subtracting the moisture, ash, protein, and fat content from the total weight of the yuja candies. The color parameters of the candies were measured using the formula CIE-L*a*b* value; L* indicates lightness, a* is redness and greenness, and b* is yellowness and blueness. The IRIS electronic eye (Alpha MOS, Toulouse, France) was used to collect visual information on the samples.

### 2.3. Melting Properties Measurement

These experiments were performed using differential scanning calorimetry (DSC) with the Q200 (TA Instrument, New Castle, DE, USA). Approximately 9.5 mg of each sample was sealed in an aluminum pan. The heating rate was 10 °C/min in the 30–200 °C range. These works were repeated three times to ensure the reproducibility of the results, and the average of traces was considered.

### 2.4. Food Texture Analysis

The texture properties of the yuja candies were conducted by a TA-XT plus C mechanical testing instrument (Stable MicroSystems Ltd., Godalming, UK). The cube-shaped yuja candy samples (approximately 15 mm (length) × 10 mm (width) × 13 mm (height)) were compacted by using a SMSP/100 probe. The measurements that were carried out were a 2 mm/s test speed, a 2 mm/s post-test speed, and a 50 g trigger force with a distance of 5 mm. The samples were compressed to 50% of their original height.

### 2.5. Analysis of Antioxidant Compound

The naringin and hesperidin components were analyzed using high-performance liquid chromatography (HPLC, Agilent, 1260B, Santa Clara, CA, USA) to investigate the analysis. The 1 g of candy powder was mixed with 50 mL of methanol (Mallinckrodt Baker, Inc., Phillipsburg, NJ, USA) and sonicated for 20 min and then filtered. An HPLC column was used, Zorbax Eclipse XDB C18 column (Agilent Technologies, Waldbronn, Germany) (4.6 mm × 250 mm, 5 μm), and acetonitrile, water and formic acid were the chemical solvents in the HPLC. Total polyphenol and flavonoid were evaluated according to methods described in the previous research [20]. The value was calculated in mg of gallic acid equivalent or rutin equivalent per g of candy using a standard curve prepared with gallic acid or rutin (Sigma-Aldrich, St. Louis, MO, USA).

### 2.6. Determination of Antioxidant Activities

2,2-Diphenyl-1-picrylhydrazyl (DPPH) radical scavenging activity was determined by the method of Blois (1958) with some modification [21]. DPPH radical scavenging activity was determined by the formula: scavenging activity (%) = [1 − (OD of the sample/OD of the blank)] × 100. 2,2′-azino-bis-(3-ethylbenzothiazoline-6-sulfonic acid) diammonium salt (ABTS, Sigma-Aldrich). Radical scavenging activity was carried out by the ABTS+ method of Biglari (2008) with some modification [22]. ABTS radical scavenging activity was calculated by the formula: scavenging activity (%) = [1 − (OD of the sample/OD of the blank)] × 100. The ferric reducing antioxidant power (FRAP) was performed by the method of Benzie and Strain (1996) with some slight modification [23].

### 2.7. Electronic Tongue Analysis

To conduct the sensory evaluation of the yuja candies, a sample was prepared by adding 100 mL of purified water to 5 g of candy and stirring at 60 °C for 1 h. The sample solution was used to measure the taste of the candies using an electronic tongue (Astree II; Alpha MOS, Toulouse, France) equipped with seven potentiometric sensors. Before sample measurements were conducted, conditioning, calibration, and diagnostics were performed using a standard sample provided by Alpha MOS. Then, each sample (100 mL) was measured for 120 s, and the average values of the last 15–20 s were used to represent the values for each sensor.

### 2.8. Statistical Analysis

Statistical analyses were performed using SPSS software version 26 (IBM Co., Chicago, IL, USA). All experiments were presented as the means ± standard error (SE). The data were analyzed with one-way analysis of variance (ANOVA) using a statistical software package (SPSS for Windows, version 26, IBM Co.) followed by Tukey’s HSD multiple-range post hoc test. Values were considered significantly different at *p* < 0.05.

## 3. Results and Discussion

### 3.1. Proximate Composition

When developing a new food product, it is common practice to analyze its physicochemical characteristics. The xylitol candies with added yuja were evaluated for proximate composition, and the results are shown in Table 1. The moisture content of the xylitol candies with added immature or mature yuja peel or pulp ranged from 0.02% to 0.03%, with no significant differences among all groups. A higher moisture content will make processed food prone to microbial and enzymatic decomposition [24]. But microbes cannot grow below a moisture content of 0.6% [25]. The xylitol candies with added yuja peel and fruit pulp developed in this study have a moisture content of 0.03% or lower, making them suitable for maintaining quality and shelf stability. The protein content also showed no significant differences among the groups, but the IYP-C group exhibited the highest value at 3.23%. The crude fat content indicated a significant difference between the candy groups with added yuja and the CON group. The carbohydrate content was lowest in the IYP-C group at 95.72%, followed by the YP-C, MYP-C, and CON groups, in that order. The ash content of a foodstuff means inorganic residue remaining after decomposition of organic matter [26]. The increase in the average ash content is related to the amount of mineral content [27]. There were significant differences in the ash contents among the groups. The ash contents ranged from 0.02 to 0.18%. The IYP-C group had the highest value (0.18%) while the YP-C group had the lowest value (0.02%). The reason for the significant improvement in the crude ash contents of the IYP-C and MYP-C groups compared to the CON or YP-C groups is due to the higher content of inorganic substances present in yuja peel. Lee et al. reported that in yuja the ash content of the peel is significantly higher than the pulp [28].

The optical properties of confectionery are a crucial factor in enhancing consumers’ overall recognition and preferences [29]. In this study, the color parameters (L*, a*, b*) of xylitol candies with added yuja were investigated and are presented in Table 2. The L* values represent brightness, and significant differences were observed in all groups. The CON group exhibited the highest brightness, followed by the YP-C, MYP-C, and IYP-C groups. This was similar to the brightness of the added yuja samples. The a* value presents color changes from red (positive) to green (negative), and the b* value presents color changes from yellow (positive) to blue (negative) [30]. The xylitol candies with added yuja showed the highest red hue in the MYP-C group, followed by the IYP-C, YP-C, and CON groups, and the same trend was observed for the yellow hue represented by the b* value. It is inferred that the xylitol candies with added yuja were influenced by the color of the added samples. Generally, xylitol candies with added yuja exhibited a yellow hue similar to the color of yuja, albeit with variations in brightness. According to the research findings, the candies, as opposed to being white, were reported to be preferred in green and yellow colors by men, whereas women tended to prefer yellow and red colors [31]. Therefore, it was determined that adding the peel or pulp of yuja to xylitol candies will enhance a preference for them by imparting yellow or red hues.

### 3.2. Melting Properties and Texture Analysis

The melting properties of hard candy influence its storage conditions, and a low melting point can adversely affect its quality [32]. Melting temperature is determined by DSC thermogram, and the maximum peak in a DSC graph changes depending on the rate of heating and the mass of the sample, but extrapolated onset temperature is not affected by such factors [33]. Therefore, in this study, we used the onset temperature as the melting temperature (T_m_), and the thermal event results of xylitol candies with added yuja peel and pulp obtained from the DSC experiments are presented in Figure 1. The T_m_ value was highest in the CON group at 93.68 ± 0.23, followed by the IYP-C (92.93 ± 0.19), MYP-C (91.86 ± 0.32), and YP-C (82.64 ± 4.78) groups, in that order. The glass transition temperature was not observed. In the study by Salaün et al., the T_m_ value of xylitol candy was 93.71 °C, which is similar to our results [34]. During storage, higher temperatures than the T_m_ of the products cause a transition from a glassy to a rubbery state, which promotes molecular mobility and, then, this change can lead to the stickiness of the candy [35]. The CON group exhibited a high T_m_ level, indicating stability during storage, and showed similar melting properties when the IYP-C and MYP-C were added. The YP-C group showed the lowest T_m_ level, which is likely due to the addition of liquid pulp during the manufacturing process. T_m_ is reported to be influenced by composition, amorphous structure, and moisture content, and we believe that in our study, the liquid of pulp likely contributed to the decrease in T_m_ during the production of the hard candy [36]. Hard candy maintains a stable glassy state when stored below its melting point, but if the storage temperature exceeds the melting point, quality deterioration accelerates [32]. Our study results suggest that the addition of immature or mature yuja peel powder or pulp to xylitol candy maintains the melting point between 82~92 °C, thereby not affecting quality stability.

The results of the texture analysis for the candies with added yuja are presented in Table 2. If the hardness and brittleness of the hard candies are low, a sticky texture occurs, making it difficult to unwrap them and resulting in an unpleasant mouthfeel, which reduces consumer acceptability [33]. In this study, the CON group exhibited the highest hardness at 44.35 kg, followed by the IYP-C (37.00), MYP-C (35.90), and YP-C (3.27) groups, in that order. Jiang et al. reported that, in xylitol candies with added flavors or flavor compounds, higher hardness results in a longer retention time of the candy and a prolonged release of flavor. Garcia Loredo and Guerrero reported that higher hardness generally leads to increased fracturability, and demonstrated that hardness has a positive relationship with fracturability [34]. Our study also demonstrated a similar trend in hardness and fracturability. When adding immature and mature yuja peel to xylitol candies, there was a tendency for texture to decrease, although this was not statistically significant. However, in the YP-C group, which had yuja pulp added, both hardness and fracturability were significantly reduced. The amount of liquid present in the hard candies during the manufacturing process is associated with a lower hardness [35]. Thus, it is believed that the liquid content of the pulp affects the texture properties such as hardness and fracturability.

### 3.3. Antioxidant Compound Contents

Polyphenols are among the most significant bioactive materials and are known for their ability to activate powerful antioxidants and free-radical scavenging activity, and to serve as hydrogen donors and reducing agents [36]. According to various studies, yuja is known to contain a rich variety of antioxidant compounds, including polyphenols and flavonoids [37]. Therefore, the antioxidant compounds of xylitol candies with added yuja were analyzed and are presented in Table 3. Total polyphenol content showed a significant difference in all candy groups, with the IYP-C group being the highest at 89.22 mg GAE/g, followed by the MYP-C, YP-C, and CON groups. According to research by Yoo et al., due to the higher antioxidant activity of yuja peel compared to its pulp, it was reported that chocolate containing yuja peel also has a higher polyphenol content compared to chocolate containing yuja pulp [38]. The flavonoid content also presented the same trend as the total polyphenol content, with the IYP-C group being the highest at 53.94 mg RE/g, which is significantly increased compared to the other groups. In particular, the IYP-C group had polyphenol and flavonoid contents that were 13.1 and 15.7 times higher, respectively, compared to the CON group. The naringin content of the MYP-C and IYP-C groups were significantly higher than that of the CON and YP-C groups. The hesperidin content was also not detected in both the CON and YP-C groups, while it significantly increased in the MYP-C and IYP-C groups to 32.70 and 33.34 mg, respectively. In addition to their antioxidant effects, naringin and hesperidin have been reported to have various other beneficial physiological activities such as anti-obesity and anti-inflammatory effects. According to research by Hwang et al., in unripe yuja, the levels of naringin and hesperidin were 504 and 695 μg/mL, respectively, whereas in mature yuja, both naringin and hesperidin decreased by more than 50% [39]. The high levels of naringin and hesperidin in the IYP-C group were, it was decided, influenced by the addition of immature yuja. Also, it has been confirmed that the antioxidant components of yuja are retained and not lost during candy processing.

### 3.4. Antioxidant Activities

**Figure 2:**
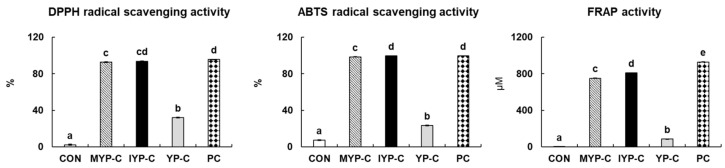
The antioxidant (DPPH, ABTS, and FRAP) activities of xylitol candies with added yuja. Values are expressed as mean ± SE (*n* = 5). Values that do not share a common letter (a–e) above the bars are significantly different among the groups using one-way ANOVA followed by Tukey’s-HSD multiple range post hoc test (*p* < 0.05). CON: xylitol candy control group, MYP-C: xylitol candy with added mature yuja peel group, IYP-C: xylitol candy with added immature yuja peel group, YP-C: xylitol candy with added yuja pulp group, DPPH: 1,1 diphenyl-2-picrylhydrazyl, ABTS: 2,2′-azino-di-2 ethyl-benzothiazoline sulfonates, FRAP: ferric reducing antioxidant power.

The natural antioxidant compounds in fruits and vegetables promote antioxidant activity such as DPPH and ABTS radical scavenging activities [40]. This antioxidant activity is known to prevent harmful free-radical oxidation in the body, enhance nutritional value, and prevent food spoilage [41]. Therefore, the antioxidant activity of xylitol candies with added yuja were evaluated by measuring DPPH and ABTS radical scavenging activities and FRAP activity and comparing them with the representative antioxidant ascorbic acid (AA). This present research showed that the DPPH radical scavenging activity of the CON, MYP-C, IYP-C, and YP-C groups were 2.35%, 92.81%, 93.83%, and 32.13%, respectively (Figure 2). In the xylitol candy groups, the IYP-C group exhibited the highest DPPH radical scavenging activity, followed by the MYP-C, YP-C, and CON groups. The DPPH solution is used in an antioxidant activity measurement method based on the principle that its radicals are neutralized and absorbance decreases when it binds with antioxidant substances [42]. Therefore, the higher the antioxidant activity, the greater the DPPH scavenging activity, and our results also showed that the IYP-C group, which is richest in polyphenols and flavonoids, exhibited the highest activity. The ABTS radical scavenging activity of the IYP-C group was the highest at 99.83% (Figure 2). Additionally, the ABTS radical scavenging activity of the CON, MYP-C, and YP-C groups were 7.35%, 98.56%, and 23.26%, respectively. ABTS radical scavenging activity is based on the principle that the ABTS free radical loses its characteristic blue color when it receives hydrogen from an antioxidant substance, transforming it into a stable compound [43]. Both DPPH and ABTS radical scavenging activities showed their highest levels in the IYP-C group compared to the other candy groups, which was similar to the ascorbic acid (positive control). The FRAP (Ferric reducing antioxidant power) assay measures the total antioxidant capacity by utilizing the reduction of the ferric tripyridyltriazine complex to ferrous tripyridyltriazine under acidic pH conditions by antioxidants [44]. The FRAP activity of the IYP-C group was significantly higher than that of the CON, MYP-C, and YP-C groups (Figure 1). Moon et al. reported that immature yuja showed a 1.5 times higher FRAP activity compared to mature yuja when comparing their antioxidant activities based on the harvesting time [19]. Yuja exhibits high antioxidant activity not only due to analyzed polyphenols and flavonoids but also because of its high content of vitamin C. Xylitol candies showed increased antioxidant activity with the addition of yuja, rich in antioxidant components. Among them, it was confirmed that the antioxidant activity was highest in the IYP-C supplemented with immature yuja, which has the highest content of polyphenols and flavonoids.

### 3.5. Sensory Properties

**Figure 3:**
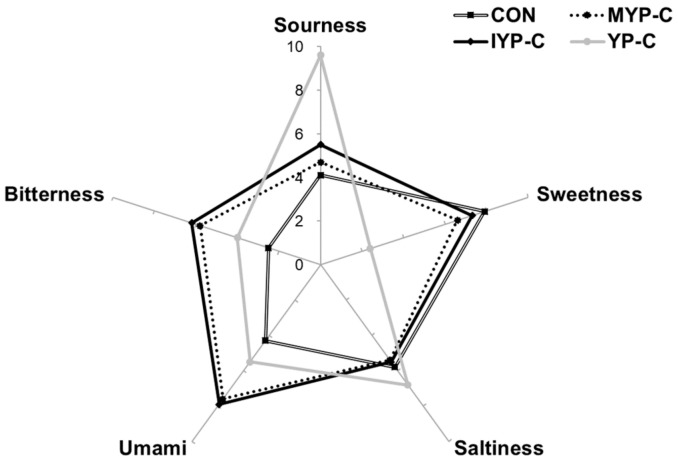
A sensory property of taste (sourness, sweetness, saltiness, umami, and bitterness) in xylitol candies with added yuja. CON: control xylitol candy group, MYP-C: xylitol candy with added mature yuja peel group, IYP-C: xylitol candy with added immature yuja peel group, YP-C: xylitol candy with added yuja pulp group.

To examine the sensory properties of xylitol candies with added yuja, sourness, sweetness, saltiness, umami, and bitterness were analyzed using an electronic tongue sensor (Figure 3). The sourness values of the CON, MYP-C, IYP-C, and YP-C were 4.1, 4.7, 5.5, and 9.6, respectively. The YP-C candies were sourer than the other candies. The sweetness values of the CON, MYP-C, IYP-C, and YP-C groups were 7.9, 6.6, 7.3, and 2.4, respectively. The CON group exhibited the highest sweetness score, followed by the IYP-C, MYP-C, and YP-C groups. Umami and bitterness in the IYP-C and MYP-C groups were significantly higher relative to the CON and YP-C groups. The saltiness value was not significantly different among the candy groups. Candy infused with yuja has a tangy, umami, and bitter taste compared to the sweet taste of xylitol candy. Choi et al. reported that the sour taste of citrus fruits significantly increased preference for them compared to jelly composed solely of xylitol by achieving a harmony with the sweetness of xylitol [45]. The addition of yuja, resulting in a refreshing taste, is expected to increase preference for xylitol candy.

## 4. Conclusions

In conclusion, the addition of immature yuja peels and mature yuja peels to xylitol candies significantly increased the levels of naringin and hesperidin, leading to enhanced antioxidant activities compared to the control and yuja pulp candies. Particularly, the IYP-C group exhibited the highest content of flavonoids and polyphenols, resulting in superior FRAP, DPPH, and ABTS+ radical scavenging activities. Additionally, the IYP-C had the highest crude ash content. Both the MYP-C and IYP-C groups displayed darker red and yellow colors compared to the CON and the YP-C groups. Sensory analysis using electronic tongue equipment revealed that the IYP-C group had elevated levels of umami, sweetness, and bitterness, while the YP-C group exhibited the highest intensity of sourness. Overall, these findings suggest that the IYP-C candies offer superior qualities in terms of antioxidant activities and physicochemical characteristics. Immature yuja especially has not been commonly used in confectionery processing so far, allowing for various applications of immature yuja in future products. This study can be utilized as a basic reference for research on processed products made from immature yuja.

## Figures and Tables

**Table 1:** The proximate compositions of the xylitol candies with added yuja.

	CON *	MYP-C	IYP-C	YP-C
Moisture (%)	0.02 ± 0.002	0.02 ± 0.002	0.03 ± 0.003	0.02 ± 0.002
Crude lipid (%)	0.45 ± 0.05 ^a^	0.96 ± 0.02 ^b^	0.84 ± 0.05 ^b^	0.83 ± 0.07 ^b^
Crude protein (%)	2.95 ± 0.12	2.45 ± 0.23	3.23 ± 0.07	3.01 ± 0.189
Crude ash (%)	0.04 ± 0.01 ^a^	0.18 ± 0.01 ^b^	0.18 ± 0.00 ^b^	0.02 ± 0.00 ^a^
Carbohydrate (%)	96.54 ± 0.16 ^b^	96.39 ± 0.24 ^ab^	95.72 ± 0.04 ^a^	96.12 ± 0.14 ^ab^

* Mean ± SE. ^a,b^ Means in the same row not sharing a common superscript letter differ significantly at *p* < 0.05. CON: candy without added yuja; MYP-C: candy with added mature yuja peel; IYP-C: candy with added immature yuja peel; YP-C: candy with added yuja pulp.

**Table 2:** The color parameters and texture analysis of the xylitol candies with added yuja.

	CON *	MYP-C	IYP-C	YP-C
L*	79.01 ± 0.14 ^d^	69.59 ± 0.08 ^b^	66.25 ± 0.02 ^a^	72.05 ± 0.05 ^c^
a*	0.90 ± 0.05 ^a^	6.57 ± 0.04 ^c^	3.87 ± 0.02 ^b^	1.04 ± 0.02 ^a^
b*	−0.57 ± 0.03 ^a^	23.50 ± 0.10 ^d^	17.55 ± 0.04 ^c^	8.90 ± 0.01 ^b^
Hardness (kg)	44.35 ± 1.03 ^c^	35.90 ± 0.49 ^b^	37.00 ± 1.02 ^b^	3.27 ± 0.22 ^a^
Fracturability (kg)	11.17 ± 0.54 ^c^	2.70 ± 0.18 ^b^	2.94 ± 0.28 ^b^	0.31 ± 0.01 ^a^

* Mean ± SE. ^a–d^ Means in the same row not sharing a common superscript letter differ significantly at *p* < 0.05. L*: lightness, a*: redness/greenness, b*: yellowness/blueness, CON: candy without added yuja; MYP-C: candy with added mature yuja peel; IYP-C: candy with added immature yuja peel; YP-C: candy added yuja pulp.

**Table 3:** The antioxidant compound contents of xylitol candies with added yuja.

	CON *	MYP-C	IYP-C	YP-C
Total polyphenol (mg GAE/g)	6.80 ± 0.21 ^a^	61.74 ± 2.93 ^c^	89.21 ± 4.04 ^d^	43.64 ± 1.85 ^b^
Total flavonoid (mg RE/g)	3.43 ± 0.60 ^a^	35.15 ± 2.82 ^c^	53.94 ± 2.78 ^d^	16.18 ± 3.05 ^b^
Naringin (mg/100 g)	0.00 ± 0.00 ^a^	13.26 ± 0.38 ^b^	13.11 ± 0.50 ^b^	0.00 ± 0.00 ^a^
Hesperidin (mg/100 g)	0.00 ± 0.00 ^a^	32.70 ± 0.59 ^b^	33.34 ± 1.72 ^b^	0.00 ± 0.00 ^a^

* Mean ± SE. ^a–d^ Means in the same row not sharing a common superscript letter differ significantly at *p* < 0.05. CON: candy without added yuja; MYP-C: candy with added mature yuja peel; IYP-C: candy with added immature yuja peel; YP-C: candy with added yuja pulp.

## Data Availability

The original contributions presented in the study are included in the article, further inquiries can be directed to the corresponding author.
